# Stressing Mitosis to Death

**DOI:** 10.3389/fonc.2014.00140

**Published:** 2014-06-04

**Authors:** Andrew Burgess, Mina Rasouli, Samuel Rogers

**Affiliations:** ^1^The Kinghorn Cancer Centre, Garvan Institute of Medical Research, Sydney, NSW, Australia; ^2^St. Vincent’s Clinical School, Faculty of Medicine, UNSW Australia, Sydney, NSW, Australia

**Keywords:** mitosis, SAC, spindle, kinetochore, checkpoint, metaphase, DNA damage, Cdk1

## Abstract

The final stage of cell division (mitosis), involves the compaction of the duplicated genome into chromatid pairs. Each pair is captured by microtubules emanating from opposite spindle poles, aligned at the metaphase plate, and then faithfully segregated to form two identical daughter cells. Chromatids that are not correctly attached to the spindle are detected by the constitutively active spindle assembly checkpoint (SAC). Any stress that prevents correct bipolar spindle attachment, blocks the satisfaction of the SAC, and induces a prolonged mitotic arrest, providing the cell time to obtain attachment and complete segregation correctly. Unfortunately, during mitosis repairing damage is not generally possible due to the compaction of DNA into chromosomes, and subsequent suppression of gene transcription and translation. Therefore, in the presence of significant damage cell death is instigated to ensure that genomic stability is maintained. While most stresses lead to an arrest in mitosis, some promote premature mitotic exit, allowing cells to bypass mitotic cell death. This mini-review will focus on the effects and outcomes that common stresses have on mitosis, and how this impacts on the efficacy of mitotic chemotherapies.

## Introduction

**Figure 1:**
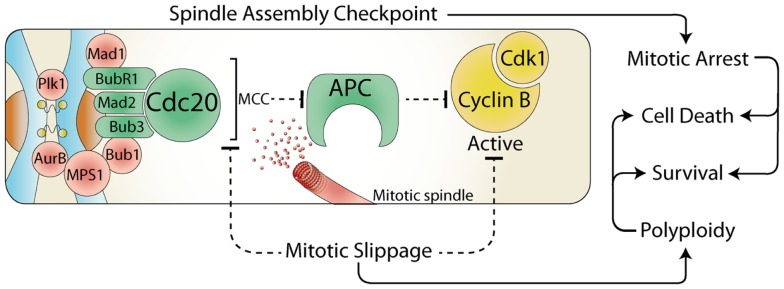
**The spindle assembly checkpoint and cell fate**. During mitosis, the constitutively active spindle assembly checkpoint (SAC) delays anaphase until all chromosomes are attached to the mitotic spindle. Any stress that prevents satisfaction of the SAC results in a prolonged mitotic arrest, which often leads to cell death. However, the SAC can be over-come by the release of Cdc20 from the mitotic checkpoint complex (MCC) or by direct inhibition of Cdk1. This mitotic slippage can result in polyploidy, increased cell survival, and provides a potential mechanism for escaping mitotic cell death.

The cell cycle is driven by the activity of the cyclin dependent kinases (Cdk), and their associated regulatory cyclin subunits. Each cell cycle phase is dependent on the sequential activation and deactivation of unique cyclin and Cdk complexes, with mitosis dependent on cyclin B bound with Cdk1 (1). To ensure the cell division process occurs with absolute fidelity, cells have developed numerous cell cycle checkpoints that delay progression in the presence of a wide variety of cellular and environmental stresses. During interphase (G1, S, and G2) stress activates checkpoints, which block cell cycle progression by increasing the translation of Cdk inhibitory proteins and activation of checkpoint kinases (Chk) that phosphorylate and inhibit Cdk (2). However, in mitosis the situation is reversed, the spindle assembly checkpoint (SAC) is on by default, which maintains high Cdk activity, thereby preventing cells from exiting mitosis. The primary role of the SAC is to block the activity of the anaphase promoting complex (APC), an E3 ubiquitin ligase responsible for targeting cyclin B1 (and many other key mitotic proteins) for degradation by the proteasome (3). This inhibition is achieved by the recruitment of several SAC proteins to the kinetochores, a protein structure located on the centromere of each chromosome (Figure 1). This localization allows the formation of the mitotic checkpoint complex (MCC) consisting of Cdc20, Mad2, Bub3, and BubR1, which then binds to and potently inhibits the APC, blocking degradation and preventing cells from entering anaphase (4). Once each kinetochore is attached to the mitotic spindle, the SAC proteins are displaced, and Cdc20 is released, allowing the APC to target proteins for degradation. However, the SAC arrest can be overcome by premature degradation of cyclin B1 (5), or direct inhibition of Cdk1 activity (6, 7) (Figure 1). This process is referred to as mitotic slippage and results in aberrant segregation of chromosomes and failure of abscission during cytokinesis, which can drive polyploidy, chromosome instability, and cancer formation (8). Therefore, during mitosis it is critical that interphase checkpoint pathways are turned off to prevent the deleterious effects of premature Cdk1 inactivation.

### Inhibition of interphase checkpoints

The inhibition of interphase checkpoints is achieved primarily by inhibition of transcription (9) and down regulation of the majority (60–80%) of protein translation (10). In addition, Cdk1 and other mitotic kinases phosphorylate and disable key effectors of interphase checkpoint pathways, providing a feedback loop that restricts this inhibition to mitosis (11).

#### Transcription

The inhibition of transcription is a critical mechanism for preventing the upregulation of Cdk inhibitor proteins, such as p21. The expression of p21 is strongly upregulated during interphase in response to a variety of cellular stresses. For example, during interphase, DNA single and double strand breaks induced by exposure to ultraviolet light (UV) or ionizing radiation (IR) respectively, results in the recruitment and activation of ataxia-telangiectasia mutated and related (ATM and ATR) kinases to the sites of damage. ATM/ATR then activate p53, which in concert with the transcription factor Sp1, increases p21 expression (12, 13). However, during mitosis the majority of proteins involved in transcription are removed from the DNA, inhibiting the production of new mRNA (14, 15). Surprisingly, transcription factors and other structural proteins can still gain access to the highly compacted chromosome structure (16), and are actively removed by mitotic kinases (17). For example, Cdk1 phosphorylates Sp1 and CUX1 resulting in their dissociation from chromatin during mitosis (18, 19), thereby preventing upregulation of p21 (20).

#### Phosphorylation

During interphase, stress often triggers a kinase phosphorylation cascade, which culminates in the inhibitory phosphorylation of the interphase Cdk. To ensure that Cdk1 is not inhibited during mitosis, these checkpoint kinases (Chk) must be inhibited. Surprisingly, Cdk1 itself disables many of these, for example, it phosphorylates Chk1/2 preventing activation by ATM/ATR (21). Furthermore, Cdk1 phosphorylation of the DNA damage signaling and repair proteins 53BP1 and BRCA1, blocks their recruitment to sites of DNA damage (22). In addition, many of these interphase Chks are repurposed and required for normal progression through mitosis. For example, Chk2 localizes to kinetochores during mitosis, stabilizing MPS1 and phosphorylating Aurora B (23). Active Aurora B then phosphorylates ATM (24), which then phosphorylates γ-H2AX and Bub1 at kinetochores (24), promoting the accumulation of Mad2 and Cdc20 (25). Consequently, ATM activity is required to ensure correct centrosome and mitotic spindle formation (26, 27).

#### Translation

The translation of mRNA into proteins is actively inhibited during mitosis (10). During interphase, the majority of mRNA is guided to ribosomes by cap-dependent translation, however as cells enter mitosis this process is repressed (9) by phosphorylation of cap-binding proteins (28). As a result, translation switches from the cap-dependent system to mRNA that contains an internal ribosomal entry site (IRES) (29). The mRNA of several important mitotic proteins contain IRES sites (30, 31), which ensures their continued translation during mitosis. In addition, the mRNA of critical mitotic factors such as cyclin B, are restricted temporally to mitosis, and locally at the mitotic spindle, by polyadenylation (32, 33).

### SAC and the response to stress in mitosis

Any stress that directly or indirectly prevents the satisfaction of the SAC prevents cells from progressing past metaphase. However, some stresses are able to deactivate the SAC and induce mitotic slippage, therefore bypassing mitotic cell death. Interestingly, mitotic slippage has been suggested as a possible mechanism for resistance to mitotic chemotherapies, in particular the microtubule poison Taxol (34). Therefore, understanding exactly how common environmental and cellular stresses affect mitosis is critical for understanding how and why some cancer cells are sensitive and others are resistant to this important class of chemotherapies.

#### DNA damage

Attempting to repair DNA during mitosis is highly dangerous for cells and can result in the fusion of telomeres, failed separation of chromatids during anaphase, and the promotion of genomic instability and cancer (22). Therefore, some have suggested that the primary mitotic response to DNA damage is to mark sites of damage (with γ-H2AX), but not to arrest in mitosis (35). Instead, damaged cells are allowed to exit to the next G1 phase where repair or death can be triggered (36). However, many cells do arrest for varying amounts of time in response to an array of DNA damaging stresses. The length of arrest roughly correlates with the level of damage, with higher levels that disrupt kinetochore–microtubule function being more efficient at blocking mitotic exit in a SAC dependent manner (37). Furthermore, a prolonged arrest can itself damage telomeres (38), suggesting that mitotic cells damage their DNA on purpose. The point of this self inflicted damage is still unclear, but it may act as a backup pathway, ensuring even minor mitotic DNA damage is fully detected in the following G1 thereby preventing defects being passed on to subsequent generations.

##### DNA decatenation

**Figure 2:**
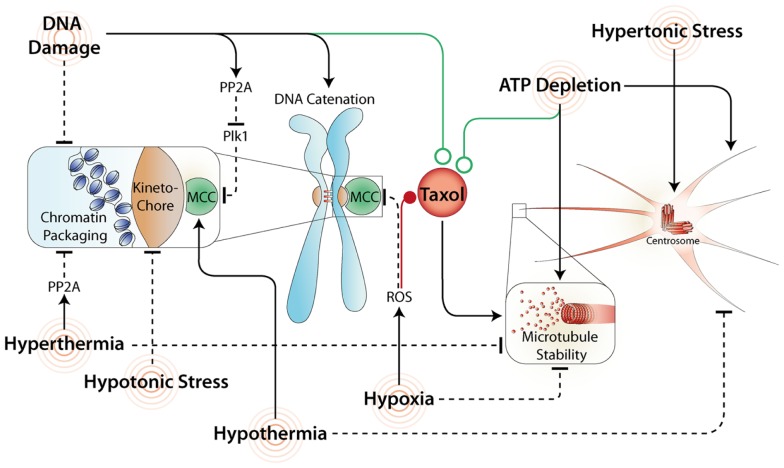
**Common stresses and their effects on mitosis and Taxol response**. A variety of stresses affect mitosis by acting directly or indirectly on the SAC. Stresses that maintain the SAC and/or increase microtubule stability often synergize (green line) with Taxol. In contrast, stresses that inhibit the MCC and/or disrupt microtubule formation commonly antagonize (red line) Taxol induced mitotic arrest and promote mitotic slippage.

During replication in interphase, sister chromatid pairs become interwound, and must be untangled prior to metaphase by decatenation, a process that requires topoisomerase II (Topo II). In addition, DNA decatenation is also required for correct chromatid and telomere separation during anaphase (39). Inhibition of Topo II during mitosis produces different mitotic responses, which are dependent on the inhibitor used, and specifically if DNA damage is produced. For example, doxorubicin creates significant levels of DNA damage (γ-H2AX foci), and consequently cells arrest in metaphase for up to 9 h (40). In contrast, ICRF-193 generally produces mild damage, and results in cells only delaying in mitosis for 1–2 h (37, 41) although ultrafine DNA bridges are formed during anaphase causing cells to fail abscission and form polyploid cells (41, 42). In all cases, the arrest during mitosis is dependent on the SAC, and is likely due to direct damage of kinetochore structure preventing stable microtubule attachments (Figure 2). For example, the delay induced by ICRF-193 requires inhibition of the APC by Mad2, but surprisingly Mad2 does not accumulate at kinetochores (35, 43). This may explain why this delay is short lived. Unfortunately, the inhibition of Topo II prior to mitosis blocks cells in G2 phase (44), consequently its use in combination with mitotic chemotherapies such as Taxol is often counter-intuitive as cells never enter mitosis and are resistant to Taxol induced death (45, 46).

##### Double strand breaks

Extensive double strand breaks during mitosis produce a strong SAC dependent arrest with cells delaying for more than 5 h over the normal 30–60 min transit time (37). Furthermore, extensive DNA damage has also been shown to inhibit the activity of Polo like kinase 1 (Plk1) (47), a mitotic kinase that plays a key role in mediating attachments between the kinetochore and mitotic spindle (48). This inhibition occurs independently of ATM (49), primarily through PP2A mediated dephosphorylation of Plk1 (50), and likely strengthens the mitotic arrest induced by extensive double strand breaks by preventing satisfaction of the SAC (Figure 2). Consequently, combining DNA damaging agents with Taxol, especially in p53 mutant cancer cells with dysfunctional interphase DNA damage checkpoints (51), may greatly enhance the amount of damage produced and promote a prolonged mitotic arrest resulting in increased levels of cell death. Accordingly, Taxol is commonly used in combination with platinum-based chemotherapeutics as a first line treatment for ovarian cancer (52), and is being trialed in combination with DNA damaging agents in several other cancer types including small cell lung cancer, melanoma, and pancreatic cancer (53–55).

##### Chromatin structure

Disruption of the mitotic chromosome architecture also produces a temporary mitotic delay. Treatment with histone deacetylase inhibitors (HDACi) prevents correct chromosome condensation and increases the access of transcription factors to the DNA, disrupting correct kinetochore formation (56). This delays the correct capture and alignment of chromosomes by the mitotic spindle, leading to SAC-dependent mitotic arrest (44, 57). However, if this damage is too severe, SAC proteins fail to remain at kinetochores, leading to silencing of the SAC and premature exit (slippage) from mitosis (58, 59). Interestingly, although HDACi have been highly successful in treatment of lymphoma, they have not been as successful with solid tumors, which could be due to SAC dysfunction (e.g., BubR1 mutation) in these cancers and an increased rate of mitotic slippage.

#### Hypoxia and oxidative stress

Reduced oxygen supply especially within the core of solid tumors results in a hypoxic environment within the tumor mass. Hypoxia is a poor prognostic factor, and correlates with resistance to radiation and many chemotherapeutic agents (60). Exposure to hypoxia during mitosis results in the rapid disruption and destabilization of microtubules (61), which delays mitotic progression. However, this arrest is unstable and cyclin B levels decrease rapidly (62), in turn inactivating Cdk1, and promoting mitotic slippage, providing an explanation for why hypoxia induces tetraploidization in melanoma (63). However, hypoxia induces a wide variety of intracellular responses including formation of reactive oxygen species (ROS), and a switch to anaerobic glycolysis resulting in decreased levels of ATP. The effects of hypoxia on mitosis are most likely due to the increased formation of ROS. In support, exposing mitotic cells to hydrogen peroxide (H_2_O_2_) to mimic ROS, induces mitotic slippage and the formation of hypertetraploid cells (64). The mechanism for this slippage is yet to be full elucidated, however, in yeast, H_2_O_2_ exposure depletes the SAC protein BubR1 from kinetochores, silencing the SAC allowing cells to exit mitosis prematurely (65). In addition, H_2_O_2_ also depolymerizes microtubules (66), which results in need for higher doses of Taxol to stabilize microtubules and induce cell death (67). These effects may explain why hypoxia reduces toxicity to Taxol in cancer cells (Figure 2). Consequently, reducing ROS with antioxidants has long been proposed as a co-treatment to enhance the effects of Taxol, with some limited success (68). The inconsistent results are likely due to the specific antioxidant used. For example, the popular dietary antioxidants Resveratrol and Fisetin (found in red wine), inhibit Cdks, induce a G2 arrest and prevent entry into mitosis (69, 70), providing an explanation for why they antagonize Taxol (71, 72). Therefore, finding methods that specifically reduce ROS without off target effects will be critical for the future success of co-treatment regimes.

#### ATP depletion

Hypoxia can also cause depletion of ATP pools, however the mitotic effects of ATP depletion are opposite to that of hypoxia and oxidative stress. Specific depletion of ATP pools with DNP, Azide, or AMP-PNP, results a rapid prolonged mitotic arrest in mammalian cells (73). ATP is needed for microtubule disassembly (74), and therefore depletion of ATP stabilizes microtubules (75). In addition, depletion of ATP activates AMP-activated protein kinase, which phosphorylates myosin regulatory light chain, and promotes astral microtubule growth (76) (Figure 2). Surprisingly, depletion of ATP also depletes Mad2 and BubR1 from kinetochores, with both proteins accumulating at spindle poles, however this does not appear to affect their ability to bind Cdc20 and inhibit the APC (77–79). Taken together, this area has significant potential for future novel therapeutic approaches, with some metabolic inhibitors already showing synergy with Taxol (80).

#### Thermal shock

Heat-shock (hyperthermia) has been commonly used as adjunctive cancer therapy to augment radiotherapy and chemotherapy, with varying levels of success (81). The initial mitotic response to acute (42°C) heat-shock is to arrest in mitosis (82). This delay is most likely SAC dependent due to effects on microtubules and centrosomes, which become permanently disorganized and destabilized upon exposure to heat (83). In addition, heat can increase the binding of the heat-shock transcription factor 2 (HSF2) to DNA during mitosis (84). HSF2 binding attracts PP2A, which dephosphorylates condensin, thereby reducing the compaction of the chromosomes (85), and acting similar to HDACi treatment (Figure 2). Consequently, the mitotic delay is only temporary, and cells rapidly reform a nuclear envelope around chromosomes, and undergo mitotic slippage (86, 87), even in the presence of Taxol (88). Hyperthermia has been shown to both antagonize (89) and synergize (88) with Taxol, with the outcome dependent on the functionality of the apoptotic pathway (90, 91).

Interestingly, like hyperthermia, cold-shock (hypothermia) has also shown some success in synergizing with radiotherapy and a variety of chemotherapeutics (92, 93). Exposure to cold induces a transient mitotic delay in cells, however cells eventually complete mitosis and segregate their chromosomes normally (94). Hypothermia reversibly destabilizes non-kinetochore microtubules (95, 96), but this still allows chromosomes to be captured by kinetochore microtubules and positioned at the metaphase plate (94). However, a reduction in microtubule dynamics and loss of astral microtubules results in reduced tension at kinetochores leading to the retention of Bub1 and BubR1 at kinetochores and a SAC-dependent mitotic delay (97). The ability of cells to recover from hypothermia and complete mitosis may explain why cold-shock can reduce the number of mitotic defects induced by chemotherapies (98), and minimize side effects (e.g., hair loss) of Taxol in cancer patients (99). However, given that the delay is transient and reversible, it also explains why co-treatment regimes have not shown any significant synergy and are unlikely to be useful for enhancing the killing of cancer cells.

#### Mechanical stress

As cells enter mitosis they transform their architecture to create a spherical shape, which is driven by changes in the actin cytoskeleton (100), and by regulation of osmotic pressure (101). The small GTPase RhoA is critical for cortical retraction during mitotic cell rounding (102). During early prophase RhoA promotes remodeling of the actin cytoskeleton, increasing the mechanical stiffness of the cell (103). Cell rounding is achieved by combining RhoA-mediated cellular rigidity with increased hydrostatic pressure inside the cell. This occurs by increasing intracellular sodium levels resulting in an influx of water (101). Failure to round up, and/or disruption of the RhoA pathway prevents mitotic exit in a SAC-dependent manner by inducing spindle pole fragmentation (104), disruption of astral microtubule organization and spindle function (105, 106) (Figure 2). Interestingly, placing cells in hypertonic solution (preventing water influx) stably arrests cells in mitosis and was originally used in the 1970s as a method for enriching mammalian cells in mitosis (107). After several hours most arrested cells die, although some escape via mitotic slippage to form polyploid cells (108). Interestingly, in yeast, hypertonic stress can promote activation of Cdc14 phosphatase (109), which then dephosphorylates Cdk substrates driving cells out of mitosis, suggesting that phosphatases can drive slippage. However, in humans the role of Cdc14 is not conserved (110), and PP2A appears to be the primary phosphatase responsible for removing mitotic Cdk1 phosphorylations (111, 112). If PP2A is directly activated in response to hypertonic stress it could promote mitotic slippage in human cells, providing a rational for future research focusing on the effectiveness of PP2A inhibitors in combination with mitotic chemotherapies.

Exposure of mitotic cells to hypotonic conditions increases water influx, rising internal pressure and a swelling of mitotic cell size, with weak hypotonic solutions arresting cells in pro-metaphase (113). However, unlike hypertonic stress, this arrest is far less stable and cells rapidly undergo mitotic slippage, characterized by chromosome decondensation, disrupted kinetochore and spindle structure, and reformation of the nuclear envelope around un-segregated chromosomes (114, 115), which all promote chromosome aberrations and polyploidy (116). The effects of hypotonic stress in combination with Taxol have not been studied in detail, however, hypotonic solutions can increase the uptake of chemotherapies in cells (117), and have shown some promise in enhancing response to platinum-based treatments (118). Consequently, it is likely that similar to hyperthermia, local hypotonic conditions could be used to enhance Taxol response in tumors with a functional apoptotic pathway.

### Conclusion/perspectives

In summary, the ability of cells to arrest during mitosis in response cellular and environmental stresses is dependent on the presence of a functional SAC, the correct suppression of transcription and translation, and critically the maintenance of Cdk1 activity. Stress that prevents the satisfaction of the SAC results in a mitotic arrest, while those stresses that disrupt Cdk1 activity or directly disable the SAC force cells to prematurely exit mitosis. Future research on the role mitotic phosphatases, such as PP2A, play in stress response and slippage will be critical for fully elucidating the mechanisms of how a specific cancer will response or can be sensitized to mitotic chemotherapies such as Taxol.

## Conflict of Interest Statement

The authors declare that the research was conducted in the absence of any commercial or financial relationships that could be construed as a potential conflict of interest.
